# C-Reactive Protein and White Blood Cell Count in Cardiogenic Shock

**DOI:** 10.3390/jcm12030965

**Published:** 2023-01-27

**Authors:** Jonas Dudda, Tobias Schupp, Jonas Rusnak, Kathrin Weidner, Mohammad Abumayyaleh, Marinela Ruka, Sascha Egner-Walter, Jan Forner, Julian Müller, Thomas Bertsch, Maximilian Kittel, Ibrahim Akin, Michael Behnes

**Affiliations:** 1 Department of Cardiology, Angiology, Haemostaseology and Medical Intensive Care, University Medical Centre Mannheim, Medical Faculty Mannheim, Heidelberg University, 68167 Mannheim, Germany; 2 European Center for AngioScience (ECAS), German Center for Cardiovascular Research (DZHK) Partner Site Heidelberg/Mannheim, 68167 Mannheim, Germany; 3 Clinic for Interventional Electrophysiology, Heart Centre Bad Neustadt, 97616 Bad Neustadt a. d. Saale, Germany; 4 Department of Cardiology and Angiology, Philipps-University Marburg, 35037 Marburg, Germany; 5 Institute of Clinical Chemistry, Laboratory Medicine and Transfusion Medicine, Nuremberg General Hospital, Paracelsus Medical University, 90419 Nuremberg, Germany; 6 Institute of Clinical Chemistry, Faculty of Medicine Mannheim, Heidelberg University, 68167 Mannheim, Germany

**Keywords:** cardiogenic shock, white blood cell count, C-reactive protein, prognosis, mortality

## Abstract

This study examines the prognostic impact of C-reactive protein (CRP) and white blood cell (WBC) counts in patients with cardiogenic shock (CS). Data regarding the prognostic impact of inflammatory biomarkers in CS are scarce. All consecutive patients with CS from 2019 to 2021 admitted to a cardiac intensive care unit (ICU) were included at one institution. Laboratory measurements were retrieved from the day of admission (i.e., day 1), as well as days 2, 3, 4, and 8. The primary endpoint was 30-day all-cause mortality. Statistical analyses included univariate *t*-tests, Spearman’s correlations, C-statistics, Kaplan–Meier, and Cox regression analyses. From a total of 240 consecutive patients admitted with CS, 55% died within 30 days. CRP levels on days 3 to 8 were associated with reliable discrimination for 30-day all-cause mortality (area under the curve (AUC): 0.623–0.754), whereas CRP on day 1 was not (AUC = 0.514). In line, CRP > 100 mg/L on day 3 (56% vs. 37%; log-rank *p* = 0.023; HR = 1.702; 95% CI 1.060–2.735; *p* = 0.028) and especially a CRP increase of at least 200% from days 1 to day 3 (51% vs. 35%; log-rank *p* = 0.040; HR = 1.720; 95% CI 1.006–2.943; *p* = 0.048) were associated with an increased risk of all-cause mortality. Furthermore, WBC on day 1 discriminated 30-day all-cause mortality (AUC = 0.605; *p* = 0.005) with an increased risk of all-cause mortality in patients admitted with WBC > 10 × 10^6^/mL (59% vs. 40%; log-rank *p* = 0.036; HR = 1.643; 95% CI 1.010–2.671; *p* = 0.045). In conclusion, WBC count on admission as well as CRP levels during the course of ICU treatment were associated with 30-day all-cause mortality. Specifically, an increase of CRP levels by at least 200% from day 1 to day 3 during the course of ICU treatment was associated with an increased risk of 30-day all-cause mortality. The present study is one of the first to describe the prognostic value of inflammatory biomarkers in consecutive all-comer CS patients treated at a cardiac ICU.

## 1. Introduction

Cardiogenic shock (CS) remains one of the leading causes of in-hospital death, with a corresponding mortality rate of approximately 50%. Despite ongoing demographic changes, acute myocardial infarction (AMI) still represents the main cause of CS, and mortality rates have been stagnating at an unsatisfactorily high level over the past decades [1,2]. Impaired ventricular function promotes end-organ damage by persistent hypoperfusion and tissue hypoxia [2,3]. In addition, pro-inflammatory pathways lead to further hypotension through several direct and indirect mechanisms (i.e., direct restraint of cardiac contractility, and reduced response to catecholamine therapy) [4]. Therefore, systemic hypoperfusion consequently leads to reflexive sympathetic activation, yet is incapable of restoring cardiac output [5]. The pro-inflammatory response in CS may correspond to the serum levels of established biomarkers and parameters such as C-reactive protein (CRP) and the white blood cell (WBC) count.

By now, blood-derived biomarkers such as lactate, cardiac troponins, and serum creatinine are routinely measured in CS patients during the course of intensive care unit (ICU) treatment. Additionally, novel biomarkers such as growth differentiating factor-15 (GDF-15), soluble suppression of tumorigenesis-2 (sST2), dipeptidyl dipeptidase 3 (DPP), galectin-3 (Gal-3), and circulating angiopoietin-2 are increasingly recognized as potential markers [6,7].

CRP is a highly conserved acute-phase inflammatory protein of the innate immune system. Depending on inflammatory cytokines, such as interleukin 6 (IL-6), CRP levels increase due to transcriptional induction in the liver [8,9]. Its main role is to activate the complement pathway in order to opsonize pathogens [9,10]. CRP levels were shown to predict prognosis in various clinical conditions such as AMI; however, their prognostic role in CS has not yet been clarified [11,12,13,14]. WBC, mainly comprising granulocytes and lymphocytes, are cells of the immune system, normally reaching between 4 × 10^9^/L and 10 × 10^9^/L. Elevated WBC counts are generally related to increased mortality in cardiovascular diseases, pursuant to several studies [15,16]. With regard to AMI patients, Klein et al. demonstrated that 30-day mortality rates were significantly higher in patients within the highest WBC quartiles, including 1892 patients with an ST-elevation myocardial infarction (STEMI) [17].

However, distinct sub-analyses investigating the prognostic value of both CRP levels and WBC counts in CS are limited, although their prognostic value was investigated in patients suffering from AMI. Therefore, this study sought to comprehensively investigate the prognostic value of CRP and WBC—reflecting the “classic” inflammatory response—during the course of ICU treatment in consecutive CS patients, irrespective of the underlying cause.

## 2. Materials and Methods

### 2.1. Study Patients, Design and Data Collection

The present study prospectively included all consecutive patients presenting with CS on admission to the cardiac ICU at the University Medical Center Mannheim, Germany, from June 2019 to May 2021. All relevant clinical data related to the index event were documented using the electronic hospital information system as well as the IntelliSpace Critical Care and Anaesthesia information system (ICCA, Philips, Philips GmbH Market DACH, Hamburg, Germany) implemented at the ICU, organizing patient data such as admission documents, vital signs, laboratory values, treatment data, and consult notes. Collected data were then pseudonymized and transferred to an electronic database (Microsoft Excel, Version 16.0, Microsoft, Redmond, WA, USA) in compliance with data protection laws. The database was password protected from unauthorized access.

Important laboratory data, ICU-related scores, hemodynamic measurements, and ventilation parameters were assessed on the day of admission (i.e., day 1), as well as on days 2, 3, 4, and 8. Furthermore, baseline characteristics, prior medical history, length of index hospital stay, data derived from imaging diagnostics, as well as pharmacological therapies were documented. Documentation of source data was performed by intensivists and ICU nurses during routine clinical care.

The present study derived from an analysis of the “Cardiogenic Shock Registry Mannheim” (CARESMA-registry), representing a prospective single-center registry including consecutive patients presenting with cardiogenic shock being acutely admitted to the ICU for internal medicine of the University Medical Center Mannheim (UMM), Germany (clinicaltrials.gov identifier: NCT05575856). The registry was carried out according to the principles of the Declaration of Helsinki and was approved by the medical ethics committee II of the Medical Faculty Mannheim, University of Heidelberg, Germany.

The medical center covers a general emergency department (ED) for emergency admission of traumatic, surgical, neurological, and cardiovascular conditions. Interdisciplinary consultation is an inbuilt feature of this 24/7 service and connects to a stroke unit, four intensive care units (ICU), and a chest pain unit (CPU) to alleviate rapid triage of patients. The cardiologic department itself includes a 24 h catheterization laboratory, an electrophysiologic laboratory, a hybrid operating room, and telemetry units. Since 2020, the University Medical Center has been a certified cardiac arrest center (CAC), including the ability to implant extracorporeal life support (ECLS) devices, such as Impella and veno-arterial extracorporeal membrane (VA-ECMO) (i.e., i-cor^®^, Xenios AG, Heilbronn, Germany, and Cardiohelp, Getinge, Gothenburg, Sweden) [18,19,20].

### 2.2. Inclusion and Exclusion Criteria, Study Endpoints

For the present study, all consecutive patients with CS and measurements of CRP and WBC count on day 1 were included. No further exclusion criteria were applied. The diagnosis of CS was determined according to the current recommendations of the Acute Cardiovascular Care Association of the European Society of Cardiology [21]. Accordingly, cardiogenic shock was defined as hypotension (SBP < 90 mmHg) for more than 30 min despite adequate filling status or the need for vasopressor or inotropic therapy to achieve SBP > 90 mmHg. Additionally, signs of end-organ hypoperfusion must be present such as oliguria with urine output < 30 mL/hour, altered mental status, cold clammy skin, and increased serum lactate > 2 mmol/L.

All-cause mortality at 30 days was documented using the electronic hospital information system and by directly contacting state resident registration offices (‘bureau of mortality statistics’). Identification of patients was verified by place of name, surname, day of birth, and registered living address. No patient was lost to follow-up with regard to all-cause mortality at 30 days.

### 2.3. Measurement of WBC Count and C-Reactive Protein

All analyses were performed in accredited laboratories under DIN ISO EN 15,189 conditions.

WBC counts were determined using dipotassium-ethylenediaminetetraacetic acid (K2-EDTA) whole blood on the hematology analyzer XN-10 on a fully automated XN-9000 platform (both Sysmex, Kobe, Japan) using a combination of flow cytometry, impedance technique, and fluorescence measurement. During the study period, two different detection systems for CRP were applied. The analyses were carried out using either serum or lithium heparin (LiHep) plasma after centrifugation in accordance with the manufacturer’s instructions. From 2019 until August 2020, CRP measurements were performed on the SIEMENS Vista Dimension 1500 (Siemens Healthineers^TM^, Erlangen, Germany) platform using nephelometric detection. The manufacturer provides an analytical measurement range of 2.90 to 190 mg/L for this assay. Thereafter, CRP levels were measured with the Siemens Atellica Solution CH 930™ (Siemens Healthineers^TM^, Erlangen, Germany). This assay is based on a latex-enhanced immunoturbidimetric detection technique. The manufacturer provides an analytical measurement range of 4.00 to 304 mg/L for this assay with a limit of detection of 4.00 mg/L. All assays were applied as stated by the respective manufacturers without further modification or validation.

Important laboratory values apart from CRP and WBC counts were measured during routine clinical care as previously published [22].

### 2.4. Statistical Methods

Quantitative data is presented as the mean ± standard error of the mean (SEM), median, and interquartile range (IQR), and ranges depending on the distribution of the data. They were compared using the Student’s *t*-test for normally distributed data or the Mann–Whitney U test for nonparametric data. Deviations from a Gaussian distribution were tested by the Kolmogorov–Smirnov test. Qualitative data are presented as absolute and relative frequencies and were compared using the chi-square test. Box plots for CRP and WBC counts were created for the comparisons of survivors and non-survivors on days 1, 2, 3, 4, and 8. On day 1, Spearman’s rank correlation for nonparametric data was used to test for the association of CRP and WBC counts with medical and laboratory parameters.

C-statistics were applied by calculation of the ROC and investigation of the corresponding AUC within the entire cohort on days 1, 2, 3, 4, and 8 in order to evaluate the prognostic performance of CRP and WBC counts with respect to the 30-day all-cause mortality. Kaplan–Meier analyses according to the median CRP levels and WBC counts on day 1 and day 3 were performed within the entire study cohort, and univariate hazard ratios (HR) were given together with 95% confidence intervals. Finally, the prognostic impact of a CRP increasement of at least 200% from day 1 to day 3 was investigated compared to patients without (“non-CRP increase”). The “non-CRP increase” group comprised patients with a CRP increase of less than 200%, stable CRP levels, or decreasing CRP levels during the first 3 days of ICU treatment. Finally, multivariate Cox regression models were developed using the “forward selection” option.

The results of all statistical tests were considered significant for *p* ≤ 0.05. SPSS (Version 28, IBM, Armonk, NY, USA) and GraphPad Prism (Version 9, GraphPad Software, San Diego, CA, USA) were used for statistics.

## 3. Results

### 3.1. Study Population

From 2019 to 2021, 273 patients with CS were admitted to our institution. After excluding 33 patients without CRP and/or WBC count measurements on day 1, the final study cohort comprised 240 CS patients. On day 1, the median WBC count was 14.73 × 10^6^/mL (IQR 10.45–18.69 × 10^6^/mL) and the median CRP was 13 mg/L (IQR 4–46 mg/L). Patients were median-aged 74 years, and most patients were males (62%) (Table 1). The rates of cardiovascular risk factors, such as arterial hypertension, diabetes mellitus, and hyperlipidemia, did not differ when stratified for 30-day survivors and non-survivors (Table 1; middle and right panels). Likewise, the rates of prior coronary artery disease (36% vs. 38%; *p* = 0.508), congestive heart failure (34% vs. 37%; *p* = 0.635), and atrial fibrillation (33% vs. 34%; *p* = 0.783) did not significantly differ among non-survivors and survivors.

Table 2 illustrates CS-related data, laboratory parameters on admission, and CS-related outcomes. AMI was the leading cause of CS, specifically in the non-survivor group (56% vs. 38%, *p* = 0.001), followed by acute decompensated heart failure (ADHF) (25% vs. 27.3%). Arrhythmia-related CS (19.4% vs. 5.3%, *p* = 0.001) was more often observed in 30-day survivors (*p* = 0.001). Besides, non-survivors were more frequently admitted with more advanced stages of CS (i.e., stage E: 65% vs. 39%; *p* = 0.001) and presented with lower left ventricular ejection fractions (LVEF) (i.e., LVEF < 30%: 58% vs. 37%; *p* = 0.004). Both in-hospital cardiac arrest (IHCA) (20.5% vs. 7.4%, *p* = 0.001) and out-hospital cardiac arrest (OHCA) (44.7% vs. 31.5%, *p* = 0.001) were more frequently observed in 30-day non-survivors. Furthermore, 30-day non-survivors were more likely to require mechanical ventilation (67% vs. 50%; *p* = 0.006) and were administered higher norepinephrine doses (median 0.2 µg/kg/min vs. 0.1 µg/kg/min; *p* = 0.001). Non-survivors had higher initial levels of lactate (4.5 mmol/L vs. 2.7 mmol/L; *p* = 0.001), creatinine (1.60 mg/dL vs. 1.36 mg/dL; 0.014), and a higher WBC count (15.61 × 10^6^/mL vs. 13.36 × 10^6^/mL; *p* = 0.005). Similarly, the international normalized ratio (INR) (1.20 vs. 1.13; *p* = 0.001) and D-dimer levels were significantly increased in non-survivors (18.19 mg/dL vs. 5.49 mg/dL; *p* = 0.005). On the contrary, baseline CRP levels did not differ between these two groups (12 mg/dL vs. 16 mg/dL; *p* = 0.698).

### 3.2. Correlation of Baseline CRP and WBC with Clinical and Laboratory Data

Table 3 demonstrates the correlations of CRP and WBC on day 1 and on day 3 with clinical and laboratory data. CRP levels correlated with bilirubin (*r* = 0.301; *p* = 0.001), procalcitonin (*r* = 0.511; *p* = 0.001), NT-pro BNP (*r* = 0.548; *p* = 0.001), and creatinine (*r* = 0.341; *p* = 0.001). Inverse correlations were observed with albumin (*r* = −0.430; *p* = 0.001) as well as mean arterial pressure (MAP) (*r* = −0.149; *p* = 0.023) and the duration of mechanical ventilation (*r* = −0.194; *p* = 0.003). The duration of mechanical ventilation displayed a positive correlation with WBC *(r* = 0.147; *p* = 0.023). Besides, the WBC count correlated with the platelet count (*r* = 0.377; *p* = 0.001), cTNI (*r* = 0.289; *p* = 0.001), and doses of catecholamines (*r* = 0.189; *p* = 0.004). Both lower age (*r* = −0.248; *p* = 0.001) and body mass index (BMI) correlated with WBC (*r* = 0.171; *p* = 0.009). Finally, there was no significant correlation perceived between CRP and WBC count (*r* = 0.089; *p* = 0.171).

On day 3, CRP correlated with WBC count (*r* = 0.255; *p* = 0.002), albumin (*r* = −0.359; *p* = 0.001), cTNI (*r* = 0.281; *p* = 0.010) and creatinine (*r* = 0.180; *p* = 0.028).

### 3.3. Prognostic Performance of CRP and WBC

**Figure 1:**
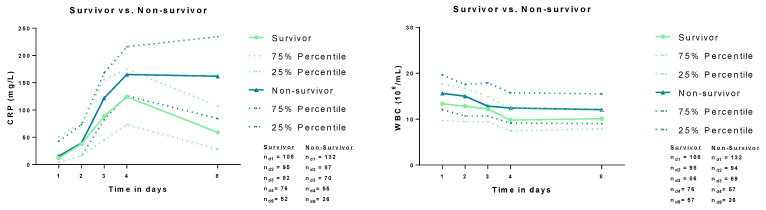
Distribution of CRP and WBC counts among survivors and non-survivors during the first 8 days after admission (i.e., on days 1, 2, 3, 4, and 8). Data is presented as median.

Overall, the risk of 30-day all-cause mortality was 55%. Figure 1 illustrates the distribution of CRP levels and WBC counts during the first week of ICU treatment on days 1, 2, 3, 4, and 8 comparing 30-day survivors and non-survivors. CRP levels did not differ among 30-day non-survivors as compared to survivors on day 1 (16 mg/L vs. 12 mg/L; *p* = 0.698) and day 2 (40 mg/L vs. 38 mg/L; *p* = 0.813). Contrarily, CRP levels differed between survivors and non-survivors on day 3 (89 mg/L vs. 120 mg/L; *p* = 0.009), day 4 (124 mg/L vs. 165 mg/L; *p* = 0.007), and day 8 (59 mg/L vs. 162 mg/L; *p* = 0.001). WBC counts were significantly higher in 30-day non-survivors compared to survivors on day 1 (15.61 × 10^6^ vs. 13.36 × 10^6^; *p* = 0.005), day 3 (12.83 × 10^6^ vs. 12.22 × 10^6^; *p* = 0.039), day 4 (12.44 × 10^6^ vs. 9.78 × 10^6^; *p* = 0.001) and day 8 (12.05 × 10^6^ vs. 10.11 × 10^6^; *p* = 0.044). On day 2, no difference in WBC was observed between survivors and non-survivors (12.82 × 10^6^ vs. 15.02 × 10^6^; *p* = 0.185).

**Figure 2:**
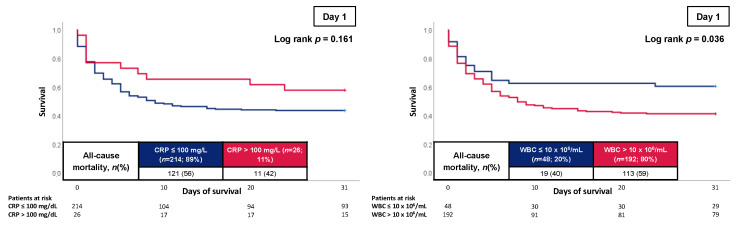
Kaplan–Meier curves of CRP and WBC count on day 1 for all-cause mortality at 30 days.

As illustrated in Table 4, the prognostic AUCs of CRP to predict all-cause mortality at 30 days were poor for day 1 (0.514; *p* = 0.700) and day 2 (AUC 0.510; *p* = 0.813), but improved on day 3 (AUC 0.623; *p* = 0.009), day 4 (AUC 0.673; *p* = 0.001), and day 8 (AUC 0.754; *p* = 0.001). In line, the WBC count displayed reliable discrimination for 30-day all-cause mortality on day 1 (AUC 0.605; *p* = 0.005), but poor discrimination on day 2 (AUC 0.572; *p* = 0.085) and day 3 (AUC 0.597; 0.039). At 30 days, all-cause mortality occurred in 59% of patients with WBC counts > 10 × 10^6^/mL on day 1 and in 40% of the patients with WBC counts ≤ 10 × 10^6^/mL (log-rank *p* = 0.036; HR = 1.643; 95% CI 1.010–2.672; *p* = 0.045) (Figure 2).

**Figure 3:**
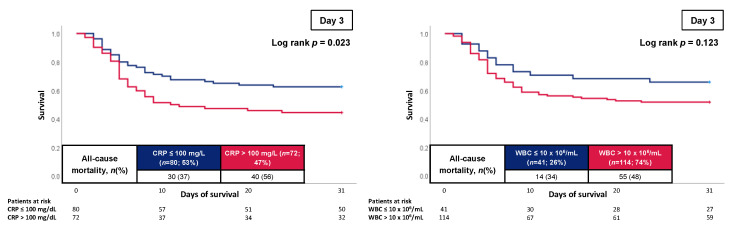
Kaplan–Meier curves of CRP and WBC count on day 3 for all-cause mortality at 30 days.

In contrast, CRP levels on day 1 were not associated with 30-day all-cause mortality (log-rank *p* = 0.161). However, on day 3, an increased 30-day all-cause mortality rate was observed in patients with CRP > 100 mg/L compared to patients with CRP levels of less or equal 100 mg/L (56% vs. 37%; log-rank *p* = 0.023; HR = 1.702; 95% CI 1.060–2.735; *p* = 0.028) (Figure 3).

**Figure 4:**
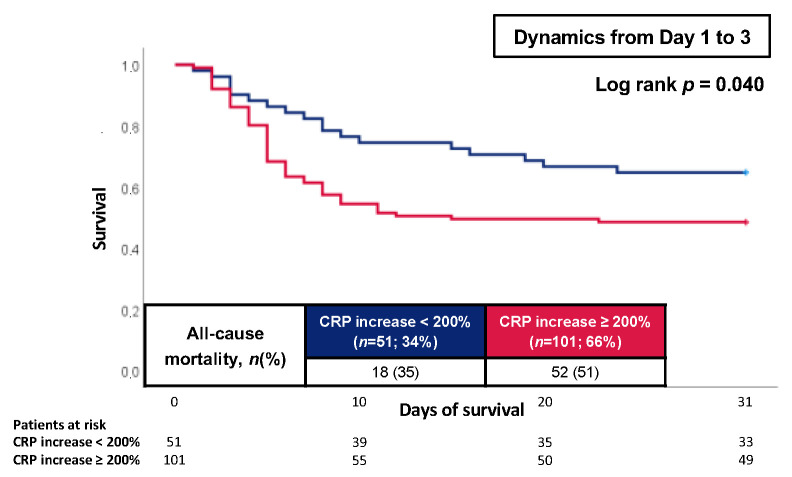
Kaplan–Meier curves of CRP dynamics from day 1 to 3 for all-cause mortality at 30 days.

Finally, a CRP increase of at least 200% in patients with CS within the first 3 days was associated with an increased risk of 30-day all-cause mortality compared to patients with a CRP increase of less than 200% (51% vs. 35%; log-rank *p* = 0.040; HR = 1.720; 95% CI 1.006–2.943; *p* = 0.048) (Figure 4).

### 3.4. Multivariate Cox Regression Models

After multivariate adjustment for potential confounders, WBC counts > 10 × 10^6^/mL on day 1 (HR 1.785; CI 1.021–3.121; *p* = 0.042) were still associated with an increased risk of 30-day all-cause mortality (HR = 1.064; CI 1.023–1.106; *p* = 0.002). In line, mechanical ventilation (HR 1.512; CI 1.005–2.277; *p* = 0.047) as well as the INR (HR 1.339; CI 1.033–1.735; *p* = 0.027) were associated with a decreased risk of 30-day all-cause mortality (Table 5). On day 3, the data exhibited an association between serum CRP levels >100 mg/L (HR 1.693; CI 1.008–2.843; *p* = 0.047) as well as INR (HR 1.615; CI 1.076–2.424; *p* = 0.021) with an enhanced risk of 30-day all-cause mortality (Table 6).

## 4. Discussion

The present study examined the short-term prognostic impact of CRP and WBC counts in consecutive CS patients admitted to a cardiac ICU. The WBC count on day 1 showed reliable discrimination of 30-day all-cause mortality (AUC 0.605; *p* = 0.005). In line, baseline WBC count > 10 × 10^6^/mL was associated with an increased risk of 30-day all-cause mortality. In contrast, admission CRP levels were not predictive. However, increased CRP levels from day 3 on showed reliable discrimination for 30-day all-cause mortality (AUC 0.623–0.754; *p* ≤ 0.009). CRP levels ≥ 100 mg/L on day 3, as well as a CRP increase of ≥200% within the first 3 days, were associated with an increased risk of 30-day mortality. Both the prognostic values of WBC on day 1 and CRP on day 3 were confirmed using multivariate Cox regression analyses.

The prognostic value of CRP and WBC in cardiovascular diseases (i.e., AMI and acute decompensated heart failure (ADHF)) was examined in numerous studies; however, only a minor portion of the patients developed CS, and distinct sub-studies investigating the prognostic role of inflammatory biomarkers in CS patients are limited.

The characterization of CS as a systemic inflammatory disorder is supported by Johansson et al. Accordingly, a proposed disease entity called shock-induced endotheliopathy (SHINE) comprises CS of different types of acute critical illnesses. Global ischemia-reperfusion drives endothelial cell damage and leads to inflammatory response [24]. According to Barron et al., high WBC counts were associated with an increased risk of 30-day all-cause mortality in 975 patients with AMI [25]. In line, WBC count was associated with higher rates of 30-day all-cause mortality, including 1892 patients with STEMI [17]. Ohlmann et al. showed an association between elevated CRP levels on admission and long-term mortality in patients after primary percutaneous interventions (PCI) in STEMI [26], which is in line with further studies [27,28]. In patients admitted with ADHF, elevated CRP levels on admission were shown to be associated with higher long-term mortality [4,29,30].

Pathophysiologically, CRP increase is predominantly upstream mediated by monocytic mediators such as IL-6 and is an activator of the classical complement pathway [9,31]. Tumor necrosis factor-alpha (TNF-alpha) and IL-1 beta are further regulatory mediators of CRP synthesis [32]. CRP increase can be triggered by various events and stimuli, such as trauma, systemic inflammatory response syndrome (SIRS), sepsis, cardiovascular, and rheumatological diseases [33]. Generally, CRP was shown to rise within 4–6 h after an inflammatory stimulus and peak after 36 to 50 h [34,35,36].

For instance, CRP levels in patients with SIRS were higher when complicated by additional infection and peaked around days 2–3 after admission [37]. This observation is in line with the findings of the present study, suggesting no association with baseline CRP (median 13 mg/dL) but improved predictive value on day 3 and thereafter.

Bahloul et al. investigated the course of biomarkers on the outcome of septic patients, and CRP appeared to peak between days 1–3. Of note, kinetics were different among survivors and non-survivors. CRP levels in survivors decreased after day 1 and remained high in non-survivors after an increase up to day 3 [38]. In line with this, Miki et al. showed that CRP levels and peaks in patients admitted with sepsis depended on the individuals’ outcomes. Among survivors, CRP levels were the highest on day 1, whereas maximum CRP levels in non-survivors were reached on day 3 [39]. WBC did not exhibit such distinct kinetics [35].

Furthermore, various studies suggest that leucocytosis can be a consequence of physical stress. In line, high serum catecholamine levels correlated with an increased WBC count after performing exhausting exercises [40,41,42,43]. Another study revealed an increase in WBC, particularly granulocytes, in resuscitated patients compared to controls (*p* < 0.001), irrespective of infection [44]. This phenomenon can be explained by endogenous and exogenous epinephrine, which stimulates granulocytes to be released into circulation after demarginating from endothelial cells [45,46]. In line with this, Benschop et al. were able to induce leucocytosis by catecholamine administration in healthy subjects [47]. These findings may also be relevant for the leukocyte counts in our registry, as cardiopulmonary resuscitation occurred in 53.4% of the patients.

The prognostic value of CRP and WBC counts regarding CS was investigated in a few studies. Sasmita et al. recently identified an increased WBC count (>11.6 × 10^6^/mL) as an independent predictor of major adverse cardiac events (MACE) in 217 patients with CS at 30 days (HR 1.894, *p* = 0.001) [48]. Most recent studies have placed more emphasis on the leukocyte-neutrophile ratio (NLR) than the overall WBC count to predict outcomes in CS. This exceeds the feasibility of large-scaled studies as the differential leukocyte count is not routinely measured. However, it was shown that higher CRP levels in patients with AMI complicated by CS increased the occurrence of MACE at 1-year. In-hospital survivors of CS revealed significantly increased CRP levels on admission compared to non-survivors, according to Akkus et al. [49]. Alongside the influence of preclinical stress and resuscitation on inflammatory markers, assistive devices may influence the inflammatory burden in patients with CS. For instance, Schrage et al. listed septic complications in around 20–30% of patients with CS plus VA-ECMO support [50].

The present study has several limitations. Due to the single-center and observational study design, results may be influenced by measured and unmeasured confounding. Furthermore, previous studies could establish a link between IL-6 and the prognosis of CS patients. However, IL-6 is not regularly measured in our institution and consequently was beyond the scope of our registry. Additionally, procalcitonin (PCT) was determined in only a small proportion of patients admitted with CS (32%); therefore, the prognostic value of PCT levels in CS was beyond the scope of the present study. For the interpretation of CRP levels, patients’ ethnicity was not registered, although ethnic background may affect CRP levels [51,52]. Besides, concomitant chronic diseases such as rheumatoid arthritis, systemic lupus erythematosus (SLE), and others that could possibly alter CRP levels have not been included in our registry. During the study period, two different assays for CRP measurement were used, which may lead to minor cofounding, although only minor differences were shown with regard to both assays [53]. Finally, CRP gene polymorphisms were shown to be associated with a marked increase in CRP and could therefore be confounders of our results [54].

In conclusion, the present study identified CRP and WBC as equally suitable prognostic markers at the early stages of cardiogenic shock, each at its preferential point in time. With respect to the observed kinetics and Kaplan–Meier analyses, we identified day 1 and day 3 as particularly worthy of predicting short-term mortality in patients with CS.

## Figures and Tables

**Table 1:** Baseline characteristics.

	All Patients (*n* = 240)		Survivor (*n* = 108)		Non-Survivor (*n* = 132)		*p*-Value
** Age ** , median; (IQR)	74	(63–81)	72	(62–80)	74	(64–81)	0.284
** Male sex, *** n * (%)	149	(62.1)	70	(64.8)	79	(59.8)	0.430
**Body mass index** (kg/m^2^), median; (IQR)	26.60	(24.20–30.25)	26.20	(24.20–29.40)	26.80	(24.50–30.50)	0.228
**Vital signs,** median; (IQR)							
Body temperature (°C)	36.0	(34.9–36.5)	36.1	(35.3–36.6)	35.8	(34.6–36.5)	**0.026**
Heart rate (bpm)	88	(71–110)	85	(69–107)	95	(72–112)	0.088
Systolic blood pressure (mmHg)	109	(93–130)	109	(93–130)	107	(91–128)	0.355
Respiratory rate (breaths/min)	20	(17–24)	19	(16–23)	20	(18–25)	0.144
**Cardiovascular risk factors,***n* (%)							
Arterial hypertension	175	(72.9)	82	(75.9)	93	(70.5)	0.343
Diabetes mellitus	98	(41.0)	39	(36.4)	59	(44.7)	0.197
Hyperlipidemia	130	(54.2)	60	(55.6)	70	(53.0)	0.696
Smoking	86	(36.0)	39	(36.4)	47	(35.6)	0.893
**Prior medical history,***n* (%)							
Coronary artery disease	89	(37.1)	41	(38.0)	48	(36.4)	0.508
Congestive heart failure	85	(35.4)	40	(37.0)	45	(34.1)	0.635
Atrial fibrillation	80	(33.3)	37	(34.3)	43	(32.6)	0.783
Chronic kidney disease	85	(35.4)	39	(36.1)	46	(34.8)	0.839
Stroke	32	(13.3)	18	(16.7)	14	(10.6)	0.169
COPD	46	(19.2)	18	(16.7)	28	(21.2)	0.373
Liver cirrhosis	8	(3.3)	5	(4.6)	3	(2.3)	0.317
Malignancy	38	(15.8)	17	(15.7)	21	(15.9)	0.972
Immunosuppression	19	(7.9)	6	(5.6)	13	(9.8)	0.220
**Medication on admission**, *n* (%)							
ACE-inhibitor	82	(37.1)	39	(36.8)	43	(37.4)	0.927
ARB	40	(18.0)	20	(18.7)	20	(17.4)	0.801
Beta-blocker	122	(55.2)	59	(55.7)	63	(54.8)	0.896
ARNI	8	(3.6)	5	(4.7)	3	(2.6)	0.395
Aldosterone antagonist	39	(17.7)	18	(17.1)	21	(18.3)	0.828
Diuretics	105	(47.3)	46	(43.4)	59	(50.9)	0.266
ASA	66	(27.5)	31	(28.7)	35	(26.5)	0.706
P2Y12-inhibitor	18	(7.5)	8	(7.4)	10	(7.6)	0.961
Statin	107	(48.2)	55	(51.9)	52	(44.8)	0.293

ACE, angiotensin-converting enzyme; ARB, angiotensin receptor blocker; ARNI, angiotensin receptor neprilysin inhibitor; ASA, acetylsalicylic acid; COPD, chronic obstructive pulmonary disease; IQR, interquartile range. Level of significance *p* < 0.05. Bold type indicates statistical significance.

**Table 2:** Shock-related data, follow-up data, and endpoints.

	All Patients (*n* = 240)		Survivor (*n* = 108)		Non-Survivor (*n* = 132)		*p* Value
**Cause of CS**, *n* (%)							
Acute myocardial infarction	115	(47.9)	41	(38.0)	74	(56.1)	
Arrhythmic	28	(11.7)	21	(19.4)	7	(5.3)	
ADHF	63	(26.3)	27	(25.0)	36	(27.3)	
Pulmonary embolism	13	(5.4)	4	(3.7)	9	(6.8)	**0.001**
Vitium	11	(4.6)	8	(7.4)	3	(2.3)	
Cardiomyopathy	7	(2.9)	4	(3.7)	3	(2.3)	
Pericardial tamponade	3	(1.3)	3	(2.8)	0	(0.0)	
**Classification of CS**, *n* (%)							
Stage A	0	(0.0)	0	(0.0)	0	(0.0)	
Stage B	6	(2.5)	6	(5.6)	0	(0.0)	
Stage C	87	(36.6)	51	(47.2)	36	(27.3)	**0.001**
Stage D	19	(7.9)	9	(8.3)	10	(7.6)	
Stage E	128	(53.3)	42	(38.9)	86	(65.2)	
**Transthoracic echocardiography**							
LVEF >55%, *n*, (%)	25	(10.4)	14	(13.0)	11	(8.3)	
LVEF 54–41%, *n*, (%)	28	(11.7)	18	(16.7)	10	(7.6)	
LVEF 40–30%, *n*, (%)	54	(22.5)	31	(28.7)	23	(17.4)	**0.004**
LVEF <30%, *n*, (%)	117	(48.8)	40	(37.0)	77	(58.3)	
LVEF not documented, *n*, (%)	16	(6.7)	5	(4.6)	11	(8.3)	
VCI (cm), median; (IQR)	1.8	(1.5–2.2)	1.8	(1.5–2.2)	1.9	(1.6–2.2)	0.345
TAPSE (mm), median; (IQR)	15	(11–18)	17	(11–20)	14	(11–17)	0.066
**Cardiopulmonary resuscitation**							
OHCA, *n* (%)	93	(38.8)	34	(31.5)	59	(44.7)	**0.001**
IHCA, *n* (%)	35	(14.6)	8	(7.4)	27	(20.5)	
Shockable rhythm, *n* (%)	171	(71.8)	78	(72.9)	93	(71.0)	0.745
Non-shockable rhythm, *n* (%)	67	(28.2)	29	(27.1)	38	(29.0)	
ROSC (min), median; (IQR)	15	(10–29)	12	(5–20)	17	(11–30)	**0.004**
**Respiratory status**							
Mechanical ventilation, *n* (%)	141	(59.5)	54	(50.0)	87	(67.4)	**0.006**
Duration of mechanical ventilation (days), mean; (IQR)	2	(1–6)	2	(0–7)	2	(1–5)	0.097
PaO2/FiO2 ratio, median; (IQR)	206	(133–338)	200	(143–356)	213	(121–337)	0.737
PaO2, mmHg, median; (IQR)	102	(78–160)	101	(78–145)	103	(77–165)	0.661
**Multiple organ support during ICU**							
Dosis norepinephrine on admission (µg/kg/min), median; (IQR)	0.1	(0.0–0.3)	0.1	(0.0–0.2)	0.2	(0.1–0.6)	**0.001**
Mechanical circulatory assist device, *n* (%)	21	(8.8)	3	(2.8)	18	(13.6)	**0.003**
**Baseline laboratory values**, (median, (IQR))							
pH	7.29	(7.22–7.37)	7.31	(7.24–7.37)	7.28	(7.19–7.37)	**0.049**
Lactate (mmol/L)	3.4	(1.7–6.9)	2.7	(1.6–4.3)	4.5	(2.2–9.7)	**0.001**
Sodium (mmol/L)	138	(136–141)	138	(136–140)	138	(136–141)	0.460
Potassium (mmol/L)	4.3	(3.8–4.9)	4.2	(3.7–4.9)	4.4	(3.9–5.0)	0.449
Creatinine (mg/dL)	1.49	(1.16–2.19)	1.36	(1.07–2.00)	1.60	(1.23–2.31)	**0.014**
Hemoglobin (g/dL)	12.4	(10.3–14.0)	12.5	(10.0–14.2)	12.4	(10.7–13.8)	0.701
WBC count (10^6^/mL)	14.73	(10.45–18.69)	13.36	(9.70–17.65)	15.61	(12.10–19.65)	**0.005**
Platelets (10^6^/mL)	222	(167–272)	218	(159–286)	224	(176–263)	0.964
INR	1.18	(1.08–1.39)	1.13	(1.05–1.32)	1.20	(1.11–1.46)	**0.001**
D-dimer (mg/L)	9.86	(2.46–32.00)	5.49	(1.99–15.74)	18.19	(3.46–32.00)	**0.005**
AST (U/L)	129	(45–312)	104	(37–205)	167	(61–488)	**0.016**
ALT (U/L)	77	(32–178)	55	(29–113)	96	(35–254)	**0.019**
Bilirubin (mg/dL)	0.62	(0.43–1.00)	0.60	(0.41–0.96)	0.65	(0.46–1.00)	0.346
Troponin I (µg/L)	0.764	(0.169–6.158)	0.335	(0.092–2.511)	1.929	(0.349–12.430)	**0.001**
NT-pro BNP (pg/mL)	4866	(971–13618)	4480	(479–12842)	5281	(1245–14104)	0.213
Procalcitonin (ng/mL)	0.30	(0.11–0.94)	0.31	(0.07–0.67)	0.28	(0.17–1.38)	0.529
CRP (mg/L)	13	(4–46)	12	(4–51)	16	(4–43)	0.698
**Primary endpoint**							
All-cause mortality at 30 days, *n* (%)	132	(55.0)	0	(0.0)	132	(100.0)	-
**Follow up data**, *n* (%)							
ICU time (days), median; (IQR)	4	(2–8)	4	(3–10)	3	(2–6)	**0.001**
Death ICU, *n* (%)	131	(54.6)	4	(3.7)	127	(96.2)	**0.001**

ADHF, acute decompensated heart failure; ALT, alanine aminotransferase; AST, aspartate aminotransferase; CRP, C-reactive protein; ICU, intensive care unit; IHCA, in-hospital cardiac arrest; INR, international normalized ratio; IQR, interquartile range; NT-pro BNP, amino-terminal pro-B-type natriuretic peptide; OHCA, out-of-hospital cardiac arrest; ROSC, return of spontaneous circulation; TAPSE, tricuspid annular plane systolic excursion; VCI, vena cava inferior; WBC, white blood cells. Level of significance *p* < 0.05. Bold type indicates statistical significance.

**Table 3:** Univariate correlations of the CRP and WBC with laboratory and clinical parameters in all patients on day 1 and day 3.

	CRP		WBC	
	r	*p*-Value	r	*p*-Value
** Day 1 **				
Age	0.085	0.191	−0.248	**0.001**
WBC count (10^6^/mL)	−0.089	0.171	−	−
Platelet count (10^6^/mL)	−0.026	0.694	0.377	**0.001**
Albumin (g/L)	−0.430	**0.001**	−0.025	0.717
Bilirubin (mg/dL)	0.301	**0.001**	−0.077	0.342
CRP (mg/L)	−	−	−0.089	0.171
Procalcitonin (ng/mL)	0.511	**0.001**	0.024	0.834
cTNI (µg/L)	0.002	0.980	0.289	**0.001**
NT-pro BNP (pg/mL)	0.548	**0.001**	−0.114	0.260
Mechanical ventilation days	−0.194	**0.003**	0.147	**0.023**
Creatinine (mg/dL)	0.341	**0.001**	0.099	0.127
Intensive care days	−0.138	**0.033**	0.076	0.243
**Day 3**				
Age	−0.078	0.339	0.117	0.146
WBC count (10^6^/mL)	0.255	**0.002**	−	−
Platelet count (10^6^/mL)	0.004	0.958	0.355	**0.001**
Albumin (g/L)	−0.359	**0.001**	−0.102	0.223
Bilirubin (mg/dL)	−0.006	0.950	−0.166	0.058
CRP (mg/L)	−	−	0.255	**0.002**
Procalcitonin (ng/mL)	0.397	**0.004**	0.123	0.395
cTNI (µg/L)	0.281	**0.010**	0.317	**0.003**
NT-pro BNP (pg/mL)	−0.011	0.943	0.158	0.324
Mechanical ventilation days	0.308	**0.001**	0.144	0.073
Creatinine	0.180	**0.028**	0.202	**0.012**
Intensive care days	0.300	**0.001**	0.074	0.363

CRP, C-reactive protein; cTNI, cardiac troponin I; NT-pro BNP, amino-terminal pro-B-type natriuretic peptide; WBC, white blood cells. Level of significance *p* < 0.05. Bold type indicates statistical significance.

**Table 4:** C-statistics to discriminate 30-day all-cause mortality by CRP and WBC.

	CRP	WBC	*p* Value *
Day 1	0.514 (0.440–0.589); *p* = 0.700	0.605 (0.533–0.677); ***p* = 0.005**	0.081
Day 2	0.510 (0.426–0.595); *p* = 0.813	0.572 (0.491–0.653); *p* = 0.085	0.298
Day 3	0.623 (0.534–0.712); ***p* = 0.009**	0.597 (0.507–0.687); ***p* = 0.039**	0.688
Day 4	0.673 (0.580–0.766); ***p* = 0.001**	0.663 (0.589–0.758); ***p* = 0.001**	0.884
Day 8	0.754 (0.633–0.875); ***p* = 0.001**	0.639 (0.499–0.778); ***p* = 0.044**	0.210

CRP, C-reactive protein; WBC, white blood cell count. Data is presented by AUC (95% CI). Level of significance *p* < 0.05. Bold type indicates statistical significance. * *p* value for the comparison of CRP and WBC according to Hanley et al. [23].

**Table 5:** Uni- and multivariate Cox regression analyses on day 1 within the entire study cohort with regard to 30-day all-cause mortality.

Variables	Univariate			Multivariate		
	HR	95% CI	*p*-Value	HR	95% CI	*p*-Value
Age	1.008	0.994–1.021	0.254	1.013	0.997–1.029	0.103
BMI (kg/m^2^)	1.014	0.983–1.046	0.386	1.005	0.970–1.042	0.774
Mechanical ventilation	1.583	1.095–2.289	**0.015**	1.512	1.005–2.277	**0.047**
Hb (mg/dL)	0.987	0.924–1.055	0.698	0.983	0.914–1.057	0.644
WBC > 10 × 10^6^/mL	1.643	1.010–2.672	**0.045**	1.785	1.021–3.121	**0.042**
Platelets (10^6^/mL)	0.999	0.997–1.001	0.424	0.999	0.997–1.001	0.458
INR	1.371	1.068–1.759	**0.013**	1.339	1.033–1.735	**0.027**
CRP > 100 mg/L	0.656	0.354–1.216	0.181	0.670	0.334–1.346	0.260

BMI, body mass index; CRP, C-reactive protein; Hb, hemoglobin; INR, international normalized ratio; WBC, white blood cell count. Level of significance *p* < 0.05. Bold type indicates statistical significance.

**Table 6:** Uni- and multivariate Cox regression analyses on day 3 within the entire study cohort with regard to 30-day all-cause mortality.

Variables	Univariate			Multivariate		
	HR	95% CI	*p* Value	HR	95% CI	*p* Value
Age	1.008	0.994–1.021	0.254	1.005	0.986–1.024	0.636
BMI (kg/m^2^)	1.014	0.983–1.046	0.386	0.983	0.935–1.034	0.506
Mechanical ventilation	1.583	1.095–2.289	**0.015**	1.309	0.746–2.300	0.348
Hb (mg/dL)	0.944	0.848–1.050	0.289	0.922	0.817–1.041	0.191
WBC > 10 × 10^6^/mL	1.565	0.870–2.815	0.135	1.571	0.842–2.931	0.155
Platelets (10^6^/mL)	0.998	0.995–1.001	0.171	0.998	0.995–1.001	0.284
INR	1.596	1.120–2.274	**0.010**	1.615	1.076–2.424	**0.021**
CRP > 100 mg/L	1.702	1.060–2.735	**0.028**	1.693	1.008–2.843	**0.047**

BMI, body mass index; CRP, C-reactive protein; Hb, hemoglobin; INR, international normalized ratio; WBC, white blood cell count. Level of significance *p* < 0.05. Bold type indicates statistical significance.

## Data Availability

The datasets used and/or analyzed during the current study are available from the corresponding author upon reasonable request.
